# Iron Deficiency and Iron Deficiency Anemia in Children with Febrile Seizure 

**Published:** 2013-01-22

**Authors:** R Fallah, B Tirandazi, S Akhavan Karbasi, M Golestan

**Affiliations:** 1 Pediatric Neurologist, Associate Professor ,Department of Pediatrics, Growth Disorders of Children Research Center, Shahid Sadoughi University of Medical Sciences and Health Services, Yazd, Iran.; 2 General Physician, Shahid Sadoughi University of Medical Sciences and Health Services, Yazd, Iran.; 3 Pediatrician, Associate Professor, Department of Pediatrics, Growth Disorders of Children Research Center , Shahid Sadoughi University of Medical Sciences and Health Services, Yazd, Iran.

**Keywords:** Seizures, Febrile, Anemia, Iron-Deficiency, Case-Control Studies

## Abstract

**Background:**

Febrile seizure (FS) is the most common childhood seizures which occur in 2-5% of children. Studies about association between iron deficiency and febrile seizure have shown contradictory results. The purpose of this study was to compare the iron status of children with first febrile seizure and healthy control group.

**Materials and Methods:**

In an analytic case-control study , iron status of 6 to 60 months old admitted children with first FS to Shahid Sadoughi Hospital from December 2011 to August 2012 was evaluated and compared with healthy age and sex matched control children whom were referred for routine health care to primary health care center of Azadshar Yazd, Iran.

**Results:**

Forty five (44%) girls and 55 boys with a mean age of 23.7 ± 14.3 months were evaluated. In children with FS , hemoglobin level (11.46 ± 1.18 g/dl vs. 11.9 ± 0.89 g/dl, p= 0.042) , serum iron levels (48.91 ± 22.96 μg/dl vs. 75.13 ± 35.57 μg/dl , p= 0.001) and serum ferritin level (38.52 ± 11.38 ng/ml vs. 54.32 ± 13.46 ng/ml, p= 0.001) were lower than in healthy children group .

Iron deficiency (48% vs. 28% , odds ratio 4.3, p=0.03) and iron deficiency anemia (22% vs. 10% , odds ratio = 3.16, p= 0.04) were more frequent in children with FS.

**Conclusion:**

Based on the result of this study, iron deficiency could be an important risk factor for development of febrile convulsion. Evaluation of iron status is encouraged to be performed in children with febrile seizure.

## Introduction

Febrile seizure (FS) is the most common type of childhood seizure which occurs in 2-5% of neurologically healthy children. FS is defined as a seizure associated with a febrile illness in the absence of central nervous system infections or acute electrolyte abnormalities in 6-60 months old children without previous afebrile seizures. FS is further classified as simple and complex types. Complex FS is defined as a seizure lasting more than 15 minutes, recurring within 24 hours or focal seizure (1).

Iron deficiency is one of the most frequent micronutrient deficiencies that affect at least one third of the population of the world. Anemia is the most common clinical manifestation of iron deficiency, but, other organs and systems may also be affected. Cognitive dysfunction, psychomotor retardation, behavioral impairments, pica, breath holding spells, restless leg syndrome and thrombosis could be associated with iron deficiency. Effect of iron deficiency in the developing brain and mechanisms such as altered development of hippocampus neurons, impairment of energy metabolism, delayed maturation of myelin, slowed visual and auditory evoked potentials and alterations in synaptic neurotransmitter systems including Norepinephrine, Dopamine, Glutamate , Gamma-Amino Butyric Acid (GABA) and serotonin may be responsible for these symptoms (2, 3).

On the other hand, fever may aggravate negative effects of iron deficiency on the brain (4).

Researches about the association between iron deficiency and febrile seizure showed conflicting results. The majority of previous studies compared ion status in febrile children who had or had not seizure. 

The purpose of this study was to compare iron status among children who had their first febrile seizure and healthy control group. This study was performed in Yazd, a city in the central region of Iran.

## Materials and Methods

In an analytic case control study, using sample size based on Z formula and confidence interval of 95% with 80% power, type one error of 5% to detect any significant difference between the two groups with a level of 0.05 , in which 50 children per group were assessed , iron status was evaluated. 

Case group consisted of 6 to 60 month old admitted children with first febrile seizure to Shahid Sadoughi Hospital Yazd, Iran from December 2011 to August 2012.

The control group consisted of healthy age and sex matched control children who were referred for routine health care to primary health care center of Azadshar.

Exclusion criteria is as follows: receiving an iron combination within the past one month , presence of any chronic systemic diseases(cardiac, renal, metabolic, malignancy, rheumatologic, etc) and having neurodevelopmental delay , previous afebrile seizure or acute central nervous system infection (meningitis , encephalitis).

Venous blood sample was obtained from the children in case and control and hemoglobin (Hb) level, Hematocrit (Hct), mean corpuscular volume (MCV), serum ferritin level , serum iron level and total iron-binding capacity (TIBC) of children were measured in the laboratory of Shahid Sadoughi Hospital.

Anemia is defined as having hemoglobin level of less than 10.5 g/dl in the 6 month to the 2 year old ones and less than 11.5 g/dl in the 2 to the 6 year old patients (5).

Iron deficiency is defined as serum ferritin level < 12 ng/ml if C-Reactive protein (CRP) is negative or 1+ , ferritin level < 30 ng/ml if CRP is ≥ 2+ , or serum iron levels of less than 22 μg/dl or transferrin saturation (a percentage calculated as serum iron concentration/TIBC×100) of less than 16 percent .The criterion had a sensitivity of 75% and specificity of 76% (5-8). 

**Statistical Analysis**

The data was analyzed using SPSS Version 17 statistical software. Chi-square test was used for data analysis of qualitative variables, and mean values were compared using independent T-test. Differences were considered significant at P-values of less than 0.05.

Informed consent was taken from patients and parents. The study has been approved by the Ethic Committee of Shahid Sadoughi University of Medical Sciences, Yazd, Iran.

## Results

Forty five girls and 55 boys with a mean age of 23.7 ± 14.3 months were evaluated. In FS group, 18 (36%) children had complex FS. Among those with complex FS, three children had multiple seizures within 24 hours, 13 had focal features and, two had prolonged convulsions. Comparison of demographic characteristic of children in both groups are shown in Table I which indicates that no statistically significant differences were observed in terms of sex distribution, mean of age and weight in both groups. 

Table II compares laboratory characteristics of children in both groups which indicates that hemoglobin level ,serum iron levels and serum ferritin level were lower in children with FS. However, no statistically significant differences were observed in terms of hematocrit, MCV and TIBC in both groups. 

Comparison of the frequency of iron deficiency and iron deficiency anemia in both groups is shown in Table III which indicates that iron deficiency and iron deficiency anemia were more frequent in children with febrile seizure. 

Table IV shows the comparison of the type of FS and the duration of seizure in children with or without iron deficiency. This indicates that in children with iron deficiency, complex FS is more frequent and seizure is longer.

**Table I:** Comparison of demographic characteristics of children in both groups. Sex distribution, mean of age and weight was not statistically different between the two groups

** Group ** **Demographic variables**		**Febrile seizure **	**Healthy control group**	**P-value**
**Sex**	**Girl**	21(42%)	24 (48%)	0.55
	**Boy**	29 (58%)	26 (52%)	
**Age in months (mean ±SD)**		22.78± 11.08	24.64± 12.28	0.52
**Weight in kilograms (mean ±SD)**		11.66 ± 3.21	12.61± 4.03	0.26

**Table II:** Comparison of laboratory characteristics of children in both groups. In febrile seizure group, hemoglobin level, serum iron levels and serum ferritin level were significantly lower

**Group ** **Laboratory ** **findings**	**Febrile seizure ** **Mean ± SD**	**Control group ** **Mean ± SD**	**P-value**
**Hemoglobin level ( g/dl )**	11.46 ± 1.18	11.9 ± 0.89	0.042
** Hematocrit ( percent) **	34.22 ± 3.23	35.08 ± 2.83	0.161
** MCV(μm3 or fl) **	75.51 ± 5.11	75.12 ± 7.37	0.766
**Serum ferritin level (ng/ml) **	38.52 ± 11.38	54.32 ± 13.46	0.001
**Serum iron levels ( μg/dl)**	48.91 ± 22.96	75.13 ± 35.57	0.001
**TIBC ( μg/dl)**	343.88 ± 72.25	341.62 ± 51.51	0.857

**Table III:** Comparison of the frequency of iron deficiency and iron deficiency anemia in both groups. Iron deficiency and iron deficiency anemia were

**Group ** **Variables**		**Febrile seizure **		**Healthy control group**		**P-value**
		**Number**	**Percent**	**Number**	**Percent**	
**Iron deficiency **	Yes	24	48	14	28	0.03
	No	26	52	36	72	
	Total	50	100	50	100	
**Iron deficiency anemia**	Yes	11	22	5	10	0.04
	No	39	78	45	90	
Total	50	100	50	100

**Table IV:** Comparison of type of FS and seizure duration in children with and without iron deficiency. In iron deficiency children, complex febrile seizure is more frequent and seizure is longer

**Group ** **Variables **		**With iron deficiency**	**Without iron deficiency**	**P-value**
**Type of febrile seizure **	**Simple**	12(37.5%)	20(62.5%)	0.04
	**Complex **	12(66.7%)	6(33.3%)	
**Duration of seizure in minute (mean ±SD)**		7.52 ± 3.77	5.17 ± 2.95	0.01

## Discussion

In the present study, iron status of children with first febrile seizure and healthy age and sex matched control children were compared. 

In the current study, among the children with FS, hemoglobin level, serum iron levels and serum ferritin level were lower than those of healthy children group . However iron deficiency (48% vs. 28%) and iron deficiency anemia (22% vs. 10%) were more frequent in children with FS which is in agreement to other studies (6,9-15). However, in Zareifar et al study in Shiraz, Iran , iron deficiency as defined serum ferritin level below 20 ng/dl was more frequent in children with FS (56.6% vs. 24.8%) and on the other hand, hemoglobin level was lower in febrile children without seizure than in FS (10). But in three other studies in Iran serum iron and plasma ferritin were higher in FS compared to febrile children without seizures (16-19). 

In Several studies from Iran, iron deficiency anemia was less frequent in children with FS, compared to febrile children without seizure (16, 18).

In Kobrinsky study in Fargo, among children with febrile seizures, iron deficiency was less frequent, but hemoglobin, hematocrit and MCV were higher. The authors suggested that iron deficiency anemia may protect the children against FS (19). 

In Talebian et al study in Kashan, Iran , risk of FS occurrence in anemic children seemed to be lower than the risk in children without anemia (20). 

Frequency of iron deficiency anemia in FS children and febrile children without seizure was not significantly different in three other Iranian studies (21, 22, 23). 

 Possible explanations for these discrepancies are differences in: age, nutritional habit, geographic area , sample size and the control group .

In the present study, iron deficiency anemia was more frequently seen among patients with complex FS, which is in agreement to another study in Turkey (24). 

Iron deficiency can alter brain synaptic neurotransmitters. Increase of glutamate excitatory neurotransmitters , decrease of GABA inhibitory neurotransmitters, decrease of monoamines and hypoxemia from iron deficiency anemia may be responsible for induction of seizure due to iron deficiency (2,3).

## Conclusion

Based on results of this study, iron deficiency and iron deficiency anemia were more frequent in children with febrile seizure and iron deficiency seems to be an important risk factor for the development of febrile convulsion. Evaluation of iron status is encouraged to be performed in children with febrile seizure.

## References

[1] Mikati MA, Kliegman RM, Stanton BF, Schor NF, St. Geme JW, Behrman RE (2011). Febrile Seizures. Nelson Textbook of Pediatrics.

[2] Yadav D, Chandra J (2011). Iron deficiency: beyond anemia. Indian J Pediatr.

[3] Johnston MV (2012). Iron deficiency, febrile seizures and brain development. Indian Pediatr.

[4] Idro R, Gwer S, Williams TN, Otieno T, Uyoga S, Fegan G (2010). Iron deficiency and acute seizures: results from children living in rural Kenya and a meta-analysis. PLoS One.

[5] Van Vranken M (2010). Evaluation of microcytosis. Am Fam Physician.

[6] Carvalho AG, Lira PI, Barros Mde F, Aléssio ML, Lima Mde C, Carbonneau MA (2010). Diagnosis of iron deficiency anemia in children of Northeast Brazil. Rev Saude Publica.

[7] Kumari PL, Nair MK, Nair SM, Kailas L, Geetha S (2012). Iron deficiency as a risk factor for simple febrile seizures--a case control study. Indian Pediatr.

[8] Morales-Ruán Mdel C, Villalpando S, García-Guerra A, Shamah-Levy T, Robledo-Pérez R, Avila-Arcos MA (2012). Iron, zinc, copper and magnesium nutritional status in Mexican children aged 1 to 11 years. Salud Publica Mex.

[9] Modaresi M, Mahmoudian T, Yaghini O, Kelishadi R, Golestani H, Tavasoli A (2012). Is Iron Insufficiency Associated With Febrile Seizure?Experience in an Iranian Hospital. J Compr Ped.

[10] Zareifar S, Hosseinzadeh HR, Cohan N (2012). Association between iron status and febrile seizures in children. Seizure.

[11] Akbayram S, Cemek M, Büyükben A, Aymelek F, Karaman S, Yilmaz F (2012). Major and minor bio-element status in children with febrile seizure. Bratisl Lek Listy.

[12] Vaswani RK, Dharaskar PG, Kulkarni S, Ghosh K (2010). Iron deficiency as a risk factor for first febrile seizure. Indian Pediatr.

[13] Sherjil A, us Saeed Z, Shehzad S, Amjad R (2010). Iron deficiency anaemia--a risk factor for febrile seizures in children. J Ayub Med Coll Abbottabad..

[14] Naveed-ur-Rehman, Billoo AG (2005). Association between iron deficiency anemia and febrile seizures. J Coll Physicians Surg Pak.

[15] Hartfield DS, Tan J, Yager JY, Rosychuk RJ, Spady D, Haines C (2009). The association between iron deficiency and febrile seizures in childhood. Clin Pediatr.

[16] Derakhshanfar H, Abaskhanian A, Alimohammadi H, ModanlooKordi M (2012). Association between iron deficiency anemia and febrile seizure in children. Med Glas Ljek komore Zenicko-doboj kantona.

[17] Bidabadi E, Mashouf M (2009). Association between iron deficiency anemia and first febrile convulsion: A case-control study. Seizure.

[18] Abaskhanian A, Vahid Shahi K, Parvinnejad N (2009). The association between iron deficiency and the first episode of febrile seizure. J Babol Univ Med Sci.

[19] Kobrinsky NL, Yager JY, Cheang MS, Yatscoff RW, Tenenbein M (1995). Does iron deficiency raise the seizure threshold?. J Child Neurol.

[20] Talebian A, Momtazmanesh N, Moosavi SGH, Khojasteh MR (2006). The relationship between anemia and febrile seizure in children under 5 years old. Iran J Pediatr.

[21] Momen A, Hakimzadeh M (2003). Case-control study of the relationship between anemia and febrile convulsion in children between 9 months to 5 years of age. Ahwaz Univ Med Sci.

[22] Salehi Omran MR, Tamaddoni A, Nasehi MM, Babazadeh H, Alizadeh navaei R (2009). Iron status in febrile seizure: a case-control study. Iranian Journal of Child Neurology.

[23] Amirsalari S, Keihani doust ZT, Ahmadi M, Sabouri A, Kavemanesh Z, Afsharpeyman S (2010). Relationship between iron deficiency anemia and febrile seizures. Iranian Journal of Child Neurology.

[24] Ozaydin E, Arhan E, Cetinkaya B, Ozdel S, Değerliyurt A, Güven A (2012). Differences in iron deficiency anemia and mean platelet volume between children with simple and complex febrile seizures. Seizure.

